# Relation of Callosal Structure to Cognitive Abilities in Temporal Lobe Epilepsy

**DOI:** 10.3389/fneur.2014.00016

**Published:** 2014-02-11

**Authors:** Christine Schneider, Christoph Helmstaedter, Eileen Luders, Paul M. Thompson, Arthur W. Toga, Christian Elger, Bernd Weber

**Affiliations:** ^1^Department of Neurology, University Hospital Bonn, Bonn, Germany; ^2^Department of Epileptology, University Hospital Bonn, Bonn, Germany; ^3^Laboratory of Neuro Imaging, Department of Neurology, University of California, Los Angeles School of Medicine, Los Angeles, CA, USA

**Keywords:** MRI, temporal lobe epilepsy, corpus callosum, full-scale IQ, intelligence, Wechsler-adult intelligence scale revised

## Abstract

The main objective of this paper is to analyze the influence of mesial temporal lobe epilepsy (mTLE) on the morphology of the corpus callosum (CC) and its relation to cognitive abilities. More specifically, we investigated correlations between intellectual abilities and callosal morphology, while additionally exploring the modulating impact of (a) side of seizure onset (b) age of disease onset. For this reason a large representative sample of patients with hippocampal sclerosis (*n* = 79; 35 males; 44 females; age: 18–63 years) with disease onset ranging from 0 to 50 years of age, and consisting of 46 left and 33 right mTLE-patients was recruited. Intelligence was measured using the Wechsler-Adult Intelligence Scale Revised. To get localizations of correlations with high anatomic precision, callosal morphology was examined using computational mesh-based modeling methods, applied to anatomical brain MRI scans. Intellectual performance was positively associated with callosal thickness in anterior and midcallosal callosal regions, with anterior parts being slightly more affected by age of disease onset and side of seizure onset than posterior parts. Earlier age at onset of epilepsy was associated with lower thickness in anterior and midcallosal regions. In addition, laterality of seizure onset had a significant influence on anterior CC morphology, with left hemispheric origin having stronger effects. We found that in mTLE, anterior and midcallosal CC morphology are related to cognitive performance. The findings support recent findings of detrimental effects of early onset mTLE on anterior brain regions and of a distinct effect particularly of left mTLE on frontal lobe functioning and structure. The causal nature of the relationship remains an open question, i.e., whether CC morphology impacts IQ development or whether IQ development impacts CC morphology, or both.

## Introduction

The main structure involved in interhemispheric information transfer in the brain is the corpus callosum (CC). The CC is the largest white matter structure connecting the two hemispheres with more than 200 million fibers (1). Clinical studies have reported abnormalities of the CC in certain neurological and psychiatric conditions, e.g., hydrocephalus (2), or Williams syndrome (3); several of these also report correlations between CC morphology and cognitive skills. They all reflect the essential importance of the CC for interhemispheric transfer of information and for cognitive abilities. Several studies in preterm born adolescents or intellectually disabled patients suggest that lower IQ levels are related to a smaller CC or white matter alterations in the CC especially in its posterior parts (4–8).

A more recent study of healthy adults by Luders et al. (1) found that IQ was positively correlated with the thickness of the posterior and (to some extent) the anterior midbody of the CC. In agreement with these findings, a study of epilepsy patients revealed that a larger posterior CC is associated with higher IQ, although the specific type of the epilepsy in these patients was not further specified (9).

Prior studies related CC and IQ measures in quite heterogeneous groups of subjects with different and quite diffuse pathologies, but our present study evaluated this relation in patients with mesial temporal lobe epilepsy (mTLE).

Mesial temporal lobe epilepsy has its onset typically in early childhood and usually becomes pharmaco-resistant over the course of time. In some cases, a surgical intervention can be regarded as an alternative treatment with a good prognosis of satisfactory seizures control in 60–80% of the patients (10). With temporo-limbic structures as origin and Ammon’s horn sclerosis (AHS) as the most common neuro-pathological correlate, its major cognitive comorbidity is an impairment of declarative episodic memory.

However, mTLE also frequently goes along with impaired intellectual abilities (11, 12). In this regard, there is now converging evidence that epilepsy patients fail to build up cognitive abilities related to frontal brain structures and that the early onset of TLE has detrimental effects on brain maturation and, consequently, cognitive development. Especially childhood and puberty seem to be critical phases in which epilepsy at its origin interferes with brain maturation and cognitive development (13, 14).

Although this is not yet well understood, several studies assume an effect of early onset mTLE on the maturation of extratemporal and especially frontal brain areas due to irradiating and interfering epileptic dysfunction (11, 15). Up to now, the impact of the onset of epilepsy on brain anatomy has been evaluated with regard to various brain structures but rarely the CC (16–19).

Building on the observations of the study of healthy adults by Luders et al. (1) the present study evaluated the morphology of the CC and its relation to IQ in patients with mTLE. Based on the fact that mTLE patients show atrophy especially in frontal brain regions, we expected the CC to be affected especially in its anterior parts and that a thinner CC would be related to greater cognitive impairments. In contrast to most prior studies of the CC, which were based either on global measures or predefined areas, we used a method with high spatial resolution to localize the presence of correlations with high anatomic precision (20). With respect to the hypothesized relationships between callosal alterations in morphology and cognitive abilities, additionally, we tested correlations between callosal morphology and full-scale IQ in a large sample of AHS patients (*n* = 79) with a wide range of intelligence quotients (IQ range: 53–128). Further, we examined the influence of hemispheric origin and the age of onset on effects of performance, measured by the Wechsler-Adult Intelligence Scale Revised (WAIS-R), on the CC.

## Materials and Methods

### Subjects

Seventy-nine right-handed mTLE-patients from the presurgical evaluation program of the Department of Epileptology at the University of Bonn were included. All patients were pharmaco-resistant and had evidence of a mesial mTLE as confirmed by MRI and electrophysiological diagnostic techniques.

The study was approved by the local ethics committee of the University of Bonn. All subjects gave their written informed consent.

In addition to intelligence scores, side of seizure onset, and age of disease onset, we also included data on gender, age, duration of disease, and education (for demographics and clinical data, see Table 1).

**Table 1:** **Descriptive statistics of WAIS-R**.

	Frequency distribution		Significance (*p*)
Gender (mean ± SD)	Female (*n* = 44) 87.08 ± 14.45	Male (*n* = 35) 93.19 ± 17.92	0.097
Age (*n* = 79) 40.42 ± 11.72	Pearson correlation coefficient = −0.50		0.659
Age of onset (*n* = 79) 15.10 ± 13.01	Pearson correlation coefficient = 0.374		0.001
Duration of epilepsy (*n* = 79) 25.32 ± 14.41	Pearson correlation coefficient = −0.378		0.001
Side of onset (mean ± SD)	Left (*n* = 46) 85.4 ± 14.92	Right (*n* = 33) 95.9 ± 16.3	0.004
Early versus late beginning	≤14 (*n* = 47) 85.43 ± 14.48	15+ (*n* = 32) 96.18 ± 16.84	0.003
Education (mean ± SD)	Education <10 years (*n* = 52) 84.02 ± 14.3	Education >10 years (*n* = 27) 100.89 ± 14.07	0.000

*WAIS-R, Wechsler-Adult Intelligence Scale Revised; *n*, number of subjects; SD, standard deviation*.

### Intelligence assessments

Intelligence scores were assessed using a short form of the revised German version of the Wechsler-Adult Intelligence Scale, the Hamburg-Wechsler-Intelligenz-Test für Erwachsene (HAWIE-R) (21). The short form comprises five subtests (picture completion, vocabulary, block design, similarities, and arithmetic) which, in a study using the total normative dataset of the German WAIS-R, best predicted the full-scale IQ. Individual IQs were determined by entering the age-corrected subtest points into a regression formula that comprised of five subtests and predicted the full-scale IQ. IQ is defined by a mean IQ of 100 with a standard deviation of 15. Here it ranged from 53 to 128. After testing for normality of distribution for all groups and subgroups, we tested for significant differences between the subgroups, respectively, correlations between collected parameters and HAWIE-R. Mean values, standard deviations, and significant differences and correlations of intelligence scores for clinical data are reported in Table 1. Because of significant differences between left and right mTLE, we further divided patients into two different groups based on their seizure onset: left (*n* = 46) and right (*n* = 33). Mean values and standard deviations of intelligence scores for the left and right TLE groups are reported in Table 2. Further we controlled correlations between full-scale IQ and callosal morphology for age of disease onset, which ranged from 0 to 50 years of age as well as for side of seizure onset.

**Table 2:** **Demographics and clinical characteristics**.

	Left TLE (*n* = 46)	Right TLE (*n* = 33)	Significance (*p*)
Gender	*f* = 31 (67.4%)	*f* = 13 (39.4%)	0.021
Age (mean ± SD)	40.6 ± 11.9 years	40.2 ± 11.7 years	0.896
Age of onset (mean ± SD)	12.3 ± 11.4 years	19.0 ± 14.2 years	0.023
WAIS-R (mean ± SD)	85.4 ± 14.9	95.9 ± 16.3	0.004
Early versus late beginning	≤14 = 29 (63.04%)	≤14 = 18 (48.28%)	0.492
Duration (mean ± SD)	28.26 ± 14.13	21.21 ± 13.97	0.031
Education	<10 years = 33 (71.7%)	<10 years = 19 (57.6%)	0.23

*f, female; WAIS-R, Wechsler-Adult Intelligence Scale Revised; *n*, number of subjects; SD, standard deviation; TLE, temporal lobe epilepsy*.

### Image acquisition and preprocessing

A T1-weighted 3D gradient echo brain MRI scan was acquired for each subject on a Siemens Avanto 1.5 T scanner (with 1 mm^3^ isotropic voxels; FOV 256 mm, matrix 256 × 256, sagittal orientation, number of slices 140, slice thickness 1 mm, TR 15.2 ms, TE 3.6 ms, flip angle 30°). All images were realigned to the ICBM-305 average brain using the software package SPM2^1^ (FIL, London, UK). This procedure corrects for different head alignment across subjects and minimizes the effect of different brain orientations on callosal measurements.

### Total brain volume analysis

The extraction of total brain volume (TBV) as well as of gray matter volumes (GMV), white matter volumes (WMV) and volume of cerebrospinal fluid (CSF) were performed with the VBM-Toolbox for SPM by Christian Gaser^2^. After positive testing volumes for normal distribution, we calculated Pearson correlation coefficient for significant correlations between intelligence measurement and TBV, GMV, WMV, and CSF separately (Table 3).

**Table 3:** **Correlation analysis of WAIS-R and brain volume**.

	Mean ± SD	Pearson correlation coefficient	Significance (*p*)
TBV (*n* = 79)	1612.53 ± 173.56	0.21	0.063
GMV (*n* = 79)	682.06 ± 82.39	0.448	0.000
WMV (*n* = 79)	442.15 ± 68.52	0.422	0.000
CSF (*n* = 79)	487.32 ± 139.42	−0.21	0.063

*WAIS-R, Wechsler-Adult Intelligence Scale Revised; TBV, total brain volume; GMV, gray matter volume; WMV, white matter volume; CSF, cerebrospinal fluid; SD, standard deviation*.

### Callosal thickness analysis

Regional callosal thickness was estimated in a three-step approach as detailed elsewhere (1, 20). Briefly, one rater (Christine Schneider) manually outlined upper and lower callosal boundaries (top and bottom) in the midsagittal section of each brain (Step I). Subsequently, the spatial average from 100 equidistant surface points representing the top and bottom traces was calculated artificially by creating a new midline segment, also consisting of 100 equidistant points (Step II). The distances between 100 corresponding surface points from this new midline to callosal top and to callosal bottom were then quantified (Step III). These regional distances indicate callosal thickness with a high spatial resolution (that is, at 100 locations distributed evenly over the callosal surface). For a more detailed description of the procedures, including illustration, see Weber et al. (22).

We calculated the correlations between individual full-scale IQ measures and callosal thickness at 100 equidistant points within the overall sample of mTLE-patients (*n* = 79). Moreover, by removing the variance associated with the age of disease onset and the hemisphere of seizure occurrence, we conducted three follow-up analyses controlling (I) for onset, (II) for hemisphere, and (III) for onset and hemisphere combined. Significance was established using a threshold of *p* ≤ 0.05 and mapped onto a group-averaged callosal surface model.

For describing significant activated areas system, we used the traditional Witelson CC classification scheme, which is mainly based on primate data (23). It should be mentioned that although most studies rely on this scheme this parcellation does not reflect specific patterns of anatomical connectivity or the texture of the CC at cellular level (24). It defines four vertical partitions, which compromise the anterior third, the anterior and posterior midbody and the posterior third. The anterior third is subdivided into the rostrum, genu, and rostral body, the posterior third into the isthmus and splenium.

## Results

### Demographics and clinical characteristics

For detailed description of the demographic and neuropsychological characteristics of the analyzed sample of 79 subjects, see Table 1.

Because of onset side differences in HAWIE-R scores, sample subjects were divided into two different groups based on seizure onset: left (*n* = 46) and right (*n* = 33). Mean values and standard deviations of intelligence scores for the left and right TLE groups are reported in Table 2. Regarding gender, age of onset, WAIS-R, and duration of epilepsy frequencies in left and right mTLE differed significantly. For age, early versus late onset, and education no significant differences were found in frequency distribution.

Further, we controlled correlations between full-scale IQ and callosal morphology for age of disease onset, which ranged from 0 to 50 years of age. More we explored any modulating impact of side of seizure.

### Total brain volume analysis

For TBV and CSF correlations with WAIS-R did not reach significance but indicate a positive relationship for TBV and a negative one for CSF. Positive but weak correlations were found between GMV and WMV. Because of absent strong correlations for GMV and WMV and absent correlations between TBV, CSF, and intelligence scores, further analyses with intracranial volume as covariates were not performed.

### Full-scale IQ

**Figure 1:**
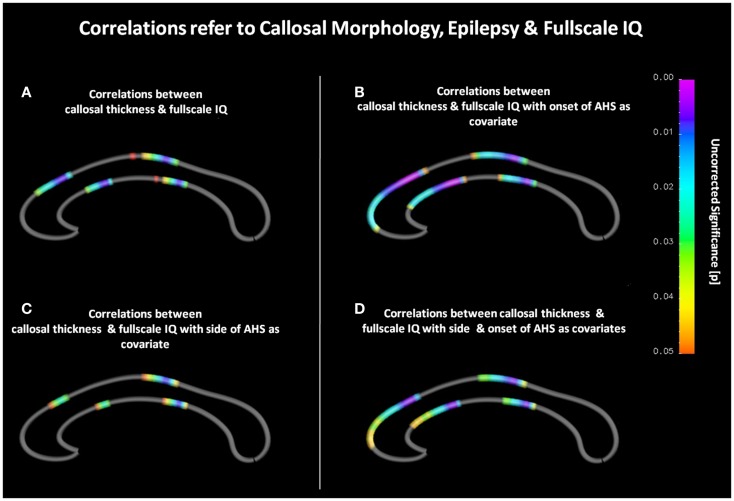
**(A)** Correlations between callosal thickness und full-scale IQ. The color bar encodes the significant *p*-values (*p* ≤ 0.05). Gray color indicates where no significant correlations were detected. The anterior callosal region is located on the left, the posterior callosal region points to the right. **(B)** Correlations between callosal thickness and full-scale IQ with age at disease onset as covariate. **(C)** Correlations between callosal thickness and full-scale IQ with hemisphere of seizure occurrence as covariate. **(D)** Correlations between callosal thickness and full-scale IQ with onset and hemisphere as covariates.

As shown in Figure 1A, we observed significant positive correlations between callosal thickness and full-scale IQ in the anterior third and posterior midbody. In the anterior third, most significant surface points (*p* ≤ 0.008) were detected at the border between the genu and the rostral body as well as between the rostral body and the anterior midbody. Another region exhibiting significant correlations was observed in a midcallosal region with the most significant area (*p* ≤ 0.008) corresponding to the posterior midbody. During the follow-up analyses, when controlling for onset, significant positive correlations between callosal thickness and full-scale IQ were evident in similar regions (i.e., anterior and posterior midbody). However, as shown in Figure 1B, effects were more pronounced and significant regions more extended, especially in anterior parts of the CC. Similar effects occurred when controlling for hemisphere as well as for age and hemisphere combined (Figures 1C,D). Significant negative correlations between callosal thickness and full-scale IQ were absent, regardless of co-varying.

## Discussion

### Correlations between callosal thickness and full-scale IQ

Callosal thickness was positively correlated with full-scale IQ in regions of the anterior body and the posterior mid callosal area (presplenial area). These findings mostly agree with results from a study of healthy adults by Luders et al. (1), where relationships between intelligence and callosal size were examined. The findings are also consistent with those from studies with patients with various neurological diseases influencing brain maturation which indicated a positive correlation between callosal area size and cognitive measurements (3, 8, 9, 16). Other studies with healthy young adults found negative correlations between cognitive abilities (25) especially in the anterior (26) and the posterior CC (27, 28), whereas prior studies on adolescents with history of very preterm birth found that poorer cognitive and neuropsychological performance were correlated with a smaller genu and posterior part of the CC, i.e., splenium and posterior midbody, or a smaller genu, isthmus, and splenium (4, 5). There is emerging evidence that negative correlations between CC and cognitive abilities are mostly found in studies with relatively young individuals indicating a dynamic process with alterations over time and influenced by a number of developmental factors (25). Regarding the healthy adults of the study by Luders et al. (1) compared to our patient group, subjects differed in mean age but were far from childhood and puberty.

However, diverging results make it obvious that relationships between CC and cognitive abilities are still not clear. Obviously, the role of the CC for information processing cannot be seen as an independent one, it is rather to ensure a fast, efficient, and orderly information transfer between various remote working cortical areas. Alterations in the morphology of the CC therefore could be induced by different brain regions and in turn effect these areas. Whether the increased callosal thickness reflects the increased number of fibers connecting both hemispheres and/or increased degree of fiber myelinization (1) still remains unexplained. Differences in examined-patient groups and methodological differences in the way the CC area is measured or subdivided into different numbers of segments may lead to inconsistent results. Also unobserved demographic variables could affect the findings.

Callosal areas more posterior are assumed to relate to the posterior association cortices, also described as the post-rolandic regions, and temporo-(post)parietal cortices. These areas are crucial for information processing and thus usually recruited in standard intelligence tests (29). Functional anatomical boundaries of callosal fibers are still imprecisely defined, so the observed correlations in the posterior midcallosal body may reflect interhemispheric connectivity between posterior association cortices required for intellectual performance and task solving.

In line with prior findings that early onset mTLE affects frontal lobe structure and intelligence, major CC deficits were observed in areas innervating the prefrontal regions, which play a key role in higher cognitive function (29, 30). With somewhat greater extent but, in essence, in agreement with Luders et al. (1), the relationships were detected in a small region at the border between the anterior third and the anterior midbody.

### Effects of age of seizure onset

When controlling for seizure onset, earlier described full-scale IQ effects on CC morphology were almost identical, but became somewhat more pronounced and extended, especially in anterior parts. It might be assumed that early seizure onset leads to greater callosal reductions especially in anterior sections. An alternative explanation could be that the effect on the CC is secondary to a dysfunctional development of the cortex. These findings are in close agreement with previous studies that found early onset effects in anterior callosal areas. Posterior callosal areas in isthmus and splenium were affected in general, regardless of disease onset, and more posteriorly than our detected full-scale IQ effects in the posterior midbody (18, 22).

In addition, recent DTI studies of mTLE-patients show lower diffusion anisotropy in frontal and temporal components of the CC, in frontoparietal lobe areas contralateral to side of seizure onset; in the isthmus and splenium of the CC, these deficits were related to earlier age of seizure onset (31, 32). Additionally, studies linking the disease, morphology and neuropsychological performance revealed correlations in mTLE patients between volume reductions in the bilateral dorsal prefrontal cortex and executive functions, or disease onset and cognitive skills required by IQ tests and related to frontal cortical areas (33, 34).

Especially white matter maturation seems to be prone to epileptogenic seizures. A DTI study of Hutchinson et al. with children with idiopathic epilepsies and limited duration of epilepsy could show microstructural white matter abnormalities suggesting a disruption in myelination processes, whereas volumetric analysis provided no evidence for WMV reductions. Additionally, animal studies demonstrate an impairment of myelin accumulation in early development in rats (14). In one recent study significant GM volume reduction in AHS patients was observed in the ipsilateral hippocampus, bilateral thalamus, insula, putamen, postcentral gyrus, and cingulated gyrus (35). In an additional DTI analysis the authors found reduced FA in the CC in the genu, body and splenium with positive correlations between ipsilateral hippocampal volume loss. The authors hypothesized that neural loss at the seizure focus causes disruption of the microstructural organization of the white matter pathways connecting the focus to distal cortical regions which then causes the atrophy in other regions. Whether those effects on the morphology of the CC are direct or indirect results of epileptic seizures is still to explore.

Altogether, our results show a clear (direct or indirect) influence of mTLE onset time on the morphology of the CC especially in anterior parts, and the WAIS-R. As these effects were found in the CC, one key component of the brain’s white matter tracts, they likely reflect a general impairment in interhemispheric connectivity. Additionally we could show a low positive correlation for WAIS-R and epilepsy onset. When comparing early versus late beginning of epilepsy disease intelligence measurements showed differences with significant lower intelligence scores in the group of early onset. However, it should also be mentioned that a likewise but negative correlation was found for WAIS-R and duration of epilepsy. Altogether results indicate a complex interaction of onset and duration of epilepsy on cognitive and brain development.

### Effects of seizure origin

When controlling for hemispheric origin of seizures, our findings agree with a study by Weber et al. (22) that revealed more pronounced anterior callosal affections in patients with left mTLE. The earlier described WAIS-R effect was still evident in both anterior and posterior callosal sections, but less pronounced and more spatially restricted in anterior sections, but the WAIS-R effect in posterior callosal sections was almost identical (22). As in the current study, previous neuropsychological studies found that patients with left mTLE, usually the dominant one, performed significantly more poorly in neuropsychological tests, such as the WAIS-R, and especially in verbal memory than those with right mTLE (13). Lateralization of mTLE seems to play a major role – mainly in verbal memory impairment – but also seems to impact multiple other cognitive modalities (36). In general, it seems as if gray matter proportions, dependent on degree of language lateralization, are more dense in the dominant cerebral hemisphere than in the non-dominant one (37). Further volumetric studies found more intense progression of gray and white matter atrophy in patients with left mTLE, and more widespread extratemporal volume reductions not only ipsilateral but also contralateral in the frontal and parietal cortex as well as reduced WMV in the left temporal lobe, CC, and bilateral prefrontal greater cortex (10, 38–40). In line with these results, further DTI analysis of white matter structures demonstrated and more extensive changes in patients with left mTLE and primarily ipsilateral changes in right mTLE with extratemporal white matter alterations in left AHS patients in the ipsilateral frontal lobe and in the genu and trunk of the CC (41, 42). Functional consequences of lateralization in general, and in mTLE patients specifically, are reported in a recent study by Pereira et al. (43). They investigated functional connectivity in right and left mTLE patients and healthy controls with left and right hippocampi as seed regions for an analysis of fiber pathways using tractography. In all groups, more pronounced patterns of functional connectivity were observed ipsilaterally, when the left hippocampus was chosen as seed as compared to right hippocampal seeds. Further, patients with left mTLE showed greater reductions of functional connectivity than right mTLE patients. However, whether functional alterations cause structural atrophy or vice versa – or whether each contributes to the other – is still an open question and further studies are needed. In concordance with structural and functional investigations, PET studies of mTLE patients indicate a lower glucose metabolism more pronounced in left lateralized mTLE with alterations in prefrontal and orbitofrontal regions, among other areas (44).

Taken together, studies differ in their methods, but their findings generally indicate more pronounced and widespread structural, functional, and metabolic alterations in left mTLE. Structural connections in the dominant (usually left) hemisphere are more dense and extensive and eventually may lead to more intensive and widespread effects of mTLE (38). Consistent with these alterations in mesiotemporal and various frontal areas, neuropsychological tests indicate not only mnestic deficits but also effects on multiple other cognitive modalities attributed to the frontal and prefrontal lobe (11, 44).

When controlling for side and onset of epilepsy, full-scale IQ effects became even more extended in the anterior CC, and slightly more significant in the posterior midbody. Overall, previously described alterations of the CC are somewhat consistent whether lateralization and onset as covariates were included or not with slightly more affected anterior parts. These findings also agree with Weber et al. (22), who revealed additional midbody and anterior CC regions affected only in left early onset mTLE-patients.

## Conclusion

Consistent with the study of healthy adults by Luders et al. (1), we observed a relationship between callosal thickness and intelligence in the anterior body as well as in the posterior midcallosal callosal area (presplenial area). These callosal regions connect cortical regions relevant for higher-order cognitive processing. Thus, our results support previous studies in by demonstrating that interhemispheric connectivity mediated by the CC in posterior association cortices as well as in the (pre-)frontal cortex are strongly involved in cognitive processes and crucial for the individual degree of intelligence. Comparing to the study of healthy adults by Luders et al. (1) it can be assumed that relationships detected in the CC in the current study in general do not reflect an mTLE specific pattern. Altogether, our findings provide evidence that CC morphology and cognitive abilities are related. Future longitudinal studies have to investigate the dynamics of this relationship and its relation to developmental and demographic factors.

Further we expected the CC to be affected especially in its anterior parts and that a thinner CC would be related to greater cognitive impairments. As expected, anterior parts were slightly more affected by age of disease onset and side of seizure onset than posterior parts. Earlier age at onset of epilepsy was associated with lower thickness in anterior and midcallosal regions. In addition, laterality of seizure onset had a significant influence on anterior CC morphology, with left hemispheric origin having stronger effects.

By recruiting mTLE-patients from our presurgical evaluation program only pharmaco-resistant patients were included in the study. Thus a control group of drug-responsive patients with few or no seizures and therefore less disease impact is missing. Moreover, additional confounding factors as duration of epilepsy, seizure frequency, and exposure to antiepileptic drugs which lead to more specificity of results should be considered in future studies. Other limitations of the current study are not included confounders as demographic variables which could affect the findings. In future studies looking at CC area and cognitive ability such parameters should be used as moderators. Also effects of the TBV were not considered and have the potential to confound findings, especially there is a known association between TBV and IQ (25, 30, 31) as well as between TBV and CC area.

Similar findings reported in prior studies comparing CC and IQ measures in quite heterogeneous groups of subjects with various neurological diseases influencing brain maturation suggests that different pathologies may lead to similar structural alterations of the CC with resulting impact on cognitive abilities. It remains unclear how specific our findings are for epilepsy. Future studies with homogeneous methods have to establish the specificity of the single diseases on alterations of the callosal morphology. However, our findings provide evidence that CC morphology can be directly or indirectly affected by certain neurological diseases (e.g., mTLE), with considerable effects of cognitive performance and as a consequence on education and future life. The causal nature of the relationship remains an open question, i.e., whether CC morphology impacts IQ development or whether IQ development impacts CC morphology, or both. Future studies are necessary to determine the complex connections between disease, neuropsychological abilities, and morphology. Moreover findings underline the importance of increased attention to be paid to the time of epilepsy onset, as well as an early diagnosis and satisfactory seizures control.

## Conflict of Interest Statement

The authors declare that the research was conducted in the absence of any commercial or financial relationships that could be construed as a potential conflict of interest.

## Footnotes

^1^http://www.fil.ion.ucl.ac.uk/spm

^2^http://dbm.neuro.uni-jena.de/vbm2_v1.06.zip
